# Cancer of the Uterine Cervix

**DOI:** 10.1186/1472-6874-4-S1-S13

**Published:** 2004-08-25

**Authors:** Eliane Duarte-Franco, Eduardo L Franco

**Affiliations:** 1 Departments of Oncology and Family Medicine, McGill University, Montreal, Canada; 2 Departments of Oncology and Epidemiology and Biostatistics, McGill University, Montreal, Canada

## Abstract

**Health issue:**

Cervical cancer is one of the most common malignant diseases of women; it is diagnosed in almost half a million women every year and half as many die from it annually. In Canada and other industrialized countries, its incidence has decreased due to cytology screening. However, invasive cases still occur, particularly among immigrant groups and native Canadian women. Although incidence of squamous cell carcinomas has decreased, the proportion of adenocarcinomas has increased because Pap cytology is ineffective to detect these lesions.

**Key findings:**

In Canada, cervical cancer will cause an estimated 11,000 person-years of life lost. In most Canadian provinces, early detection is dependent on opportunistic screening. Primary prevention can be achieved through health education (sexual behavior modification) and vaccination to prevent infection from Human Papillomavirus (HPV). The initial results from vaccination trials are encouraging but wide scale use is more than a decade away.

**Data gaps and recommendations:**

Most cases of cervical cancer occur because the Pap smear was either false negative, was not done or not done often enough. Appropriate recommendations and guidelines exist on implementation of cytology-based programs. However, most Canadian women do not have access to organized screening. Further research is needed to 1) evaluate automated cytology systems; 2) define appropriate management of precursor lesions and 3) deliver definitive evidence of HPV testing efficacy in long-term follow-up studies with invasive cancer as an outcome and 4) provide Canadian data to justify augmenting or modifying current programs to use HPV testing in secondary triage of equivocal Pap smears.

## Background

Cervical cancer is a malignant neoplastic disease that tends to begin slowly when there is a disruption of the cervical epithelium, near the squamocolumnar junction of the uterine cervix. Initially, this pre-invasive process is limited to the cervical epithelium and is known variably as cervical intraepithelial neoplasia (CIN), according to the classification scheme mostly used in histopathology, or as squamous intraepithelial lesion (SIL), as per the classification system favoured for cytopathological diagnosis. Low-grade SIL (LSIL) (equivalent to CIN 1) and high-grade SIL (HSIL, equivalent to CIN 2 and 3) are invariably asymptomatic and can be detected through cytological examination using the Papanicolaou technique (the Pap test). Their presence is confirmed by magnification during colposcopic examination and by biopsy. If left untreated, LSIL may become HSIL, and the latter may eventually extend to the full thickness of the cervical epithelium, a condition that is recognized as cervical carcinoma in situ (CIS).

Subsequently, the disease may become invasive. This process may take a decade or longer. There are two main histological types of invasive cancer: squamous cell carcinomas and adenocarcinomas. The invasive lesion may metastasize to nearby pelvic and distant lymph nodes and other body sites. The symptoms and signs in most women with invasive cervical cancer include post-coital bleeding, recurrent bladder infections and ulcers on the cervix. Pressure against nerve trunks and the sacral plexus produces persistent pain. As soon as lymph node metastasis occurs the disease worsens considerably.

The women most at risk of this disease in North America are Native populations, Black women, the Hispanic population and recent immigrant groups. Screening programs may not be reaching these women, and when cervical cancer is eventually diagnosed there may be barriers in the way of speedy access to the most recent and effective treatment methods. Women of low socio-economic status also face a less favourable outcome.

## Methods

Data on incidence rates and rates of mortality from cervical cancer in Canada were obtained from Statistics Canada and the Ministère de la santé et des services sociaux du Québec, and in the United States from the Surveillance, Epidemiology, and End Results (SEER) Program. Other epidemiologic and risk factor information was found in published surveillance and research reports.

## Results

### Cervical Cancer in Canada

Canada has been a pioneer among Western countries in adopting wide-scale cervical cancer screening with the Pap test, and this has greatly contributed to its current status as a country with one of the lowest incidence rates of the disease worldwide. Cervical cancer is expected to be newly diagnosed in an estimated 1,400 Canadian women in 2003 and will claim approximately 420 lives in that period.[1]

#### Incidence and Mortality

**Figure 1:**
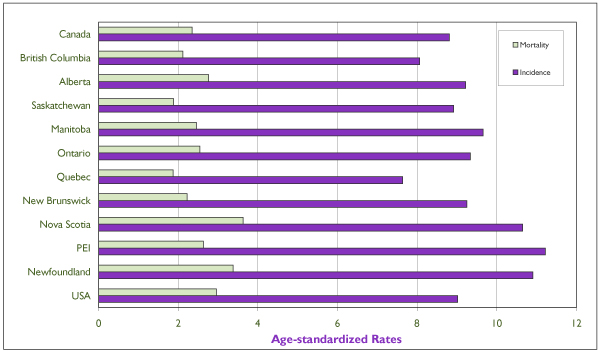
Average (Period 1994–1998), Annual Age-Standardized Incidence and Mortality Rates of Invasive Cervical Cancer for Individual Canadian Provinces, for Canada, and for the United States All rates refer to numbers of new cases or deaths per 100,000 and are standardized according to the Canadian population of 1991. Incidence data for the U.S. refer to the nine main state or metropolitan area registries belonging to the Surveillance, Epidemiology, and End Results (SEER) Program from the Cancer Surveillance Research Program of the U.S. National Cancer Institute (NCI), and mortality data refer to the entire U.S.population. Source: Statistics Canada and SEER database.[4]

Figure 1 shows the annual incidence and mortality rates of cervical cancer (averages for the latest five-year reporting period of 1994–1998) for Canada, for all Canadian provinces, and for the United States, as a comparison. The provinces with the highest incidence were Nova Scotia, Newfoundland and Labrador and P.E.I., with rates exceeding 10 per 100,000 women; Nova Scotia and Newfoundland and Labrador also showed the highest mortality rates among the provinces. Quebec and British Columbia had the lowest incidence rates. Saskatchewan and Quebec were the only two provinces with mortality rates below 2 per 100,000 per year.

#### Changes Over Time

**Figure 2:**
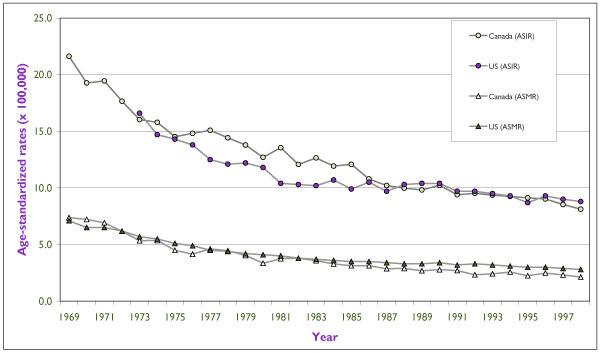
Trends in Incidence and Mortality Rates for Invasive Cervical Cancer for Canada (Since 1969) and for the United States (Since 1973). Rates are per 100,000 and are standardized according to the Canadian population of 1991. Incidence data for the United States refer to the nine main SEER registries and mortality data refer to the entire U.S. population. Source: Statistics Canada and SEER database.[4]

As a general rule, incidence and mortality rates of cervical cancer have declined in North America during the last 50 years in consequence of the increased availability of Pap smear screening programs and probably also the decline in fertility rates during the last half-century. Figure 2 shows the time trends in age-standardized incidence and mortality of invasive cervical cancer for Canada and for the United States since 1969. Although in the last 10 years incidence rates have been very similar between the two countries, Canadian women have a slight, yet consistent, advantage over their U.S. counterparts with respect to mortality from cervical cancer.

**Figure 3:**
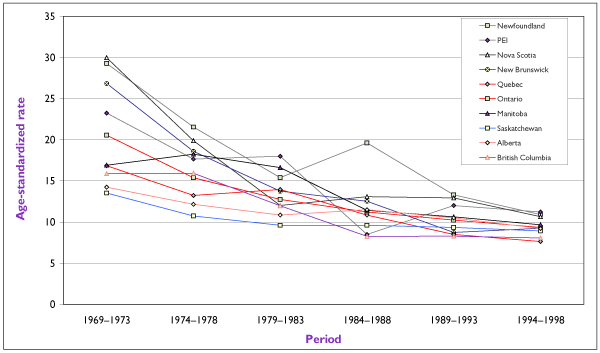
Trends in Incidence Rates for Invasive Cervical Cancer for CanadianProvinces Rates are standardized according to the Canadian population of 1991 and are presented as annual averages (per 100,000) for successive five-year periods. Source: Statistics Canada.

The decline in incidence has varied by province, as shown in Figure 3. Some of the most dramatic decreases were seen in the Atlantic provinces, in particular Nova Scotia and P.E.I. The decrease in rates is not as pronounced in provinces such as Saskatchewan and Alberta, which have had the benefit of low cervical cancer rates during most of the last 30 years. Although the range of rates among provinces in 1969–1973 was wide (13 to 30 per 100,000), the differences have levelled off to 7.6–11.2 per 100,000 in the most recent five-year period of 1994–1998.

**Figure 4:**
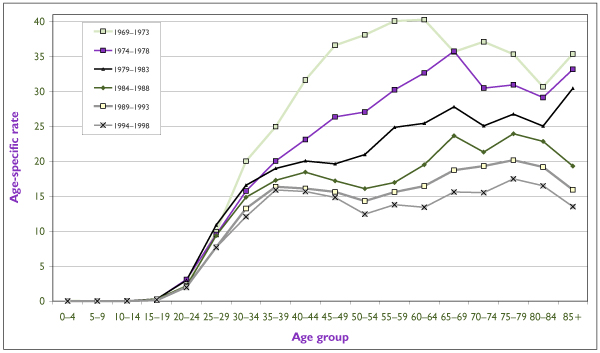
Trends in Age-Specific Incidence Rates for Invasive Cervical Cancer inCanada Since 1969 Rates are presented as annual averages (per 100,000) for successive five-year periods. Source: Statistics Canada.

The decline in cervical cancer incidence in Canada can be further examined by comparing age-specific rates by successive five-year periods (Figure 4). The reduction in incidence in the late 1960s and early 1970s was most pronounced among women between the ages of 45 and 65. With successive periods, the net reduction shifted to older women. Two incidence peaks have emerged since the mid-1980s, one in women 35 to 44 years of age and another in women aged 75 and over. The first peak seems to be shifting to younger ages, possibly as a consequence of increased opportunity for early diagnosis even among invasive cancer cases. Since cervical cancer is linked to multiple sexual partners, one cannot rule out the possible contribution of a cohort effect due to the major changes in social mores that began to occur in the late 1960s and that led to increased opportunities for exposure to human papillomavirus (HPV) infection at an early age (see "The Role of HPV" below).

Most cervical cancers are squamous cell carcinomas. While the decline of cervical cancer incidence applies to this histological type, adenocarcinomas have actually increased in incidence over the last 30 years in most Western countries. The latter used to account for about 5% of all invasive cervical cancers in the early 1970s and now constitute approximately 20% of all such neoplasms.

### The Global Burden of Cervical Cancer

Cervical cancer is one of the most common malignant diseases of women. An estimated 471,000 new cases of invasive cervical carcinoma are diagnosed annually worldwide, with a disproportionately heavy burden of the disease (380,000 new cases) occurring in developing countries. In 2000 there were an estimated 233,000 deaths from cervical cancer worldwide.[2] Its worldwide incidence represents nearly 10% of all female cancers, and it is the third most common anatomic location among women, afterbreast and colorectal cancer.[3] The highest risk areas are in Central and South America, southern and eastern Africa, and the Caribbean, where average incidence rates exceed 40 per 100,000 women per year.[2] Cervical cancer is the most common female neoplasm in regions such as eastern Africa and the Caribbean, accounting for 20% to 30% of all malignancies.[2] The risk in western Europe and NorthAmerica is considered relatively low, at fewer than 10 new cases annually per 100,000 women, whereas in high-incidence countries the rates are 10 times greater than this and the cumulative lifetime risk can approach 10%.

**Figure 5:**
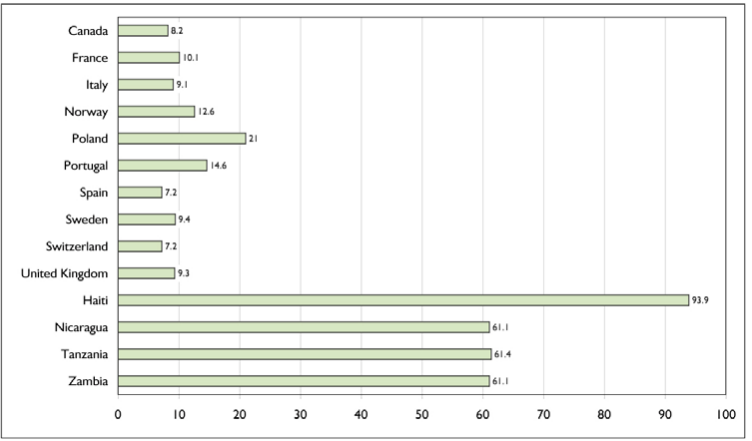
Annual Age-Standardized Incidence Rates of Invasive Cervical Cancer (Projection for 2000) for Canada and Selected European, Latin American, Caribbean and African Countries. Rates are per 100,000 and are standardized according to the world population of 1960. Source: Globocan, International Agency for Research on Cancer.[2]

Figure 5 shows cervical cancer incidence and mortality rates for Canada and selected European countries as projected for the year 2000. Among the countries chosen for comparison, only Spain and Switzerland have lower rates of the disease than Canada. The rate of mortality from cervical cancer in Canada is also lower than in most European countries.

### Vulnerable Subgroups

Cervical cancer takes a particularly heavy toll in North American Native populations, Black women, and Hispanic minorities. Black women in the United States have about a 50% greater risk of acquiring cervical cancer than white women but more than twice the risk of dying from the disease.[4] Ethnic disparities in cervical cancer risk are not as readily monitored by provincial tumour registries as they are in the SEER program in the United States. Such comparisons in Canada rely on occasional surveys. Among the Canadian Inuit, cervical cancer accounts for nearly 15% of all female cancers, a relative frequency that is comparable to that seen in developing countries. The proportion is even greater among registered Indians in Saskatchewan, at 29%, which results in an age-standardized rate six times as high as the national average.[5] Also of concern is the fact that recent immigrant groups to Canada appear to have lower rates of Pap testing and may not have been reached by the health promotion approaches used by the provinces to provide Pap smear screening and appropriate management of precursor lesions. This may eventually lead to further disparities among ethnic groups in rates of incidence and mortality.

### Survival

An average 26 years of life are lost per woman dying of cervical cancer in Canada. On the basis of this average loss of life and of the number of deaths each year in Canada, it is estimated that cervical cancer caused an estimated 11,000 person-years of life lost in 1997.[6]

**Figure 6:**
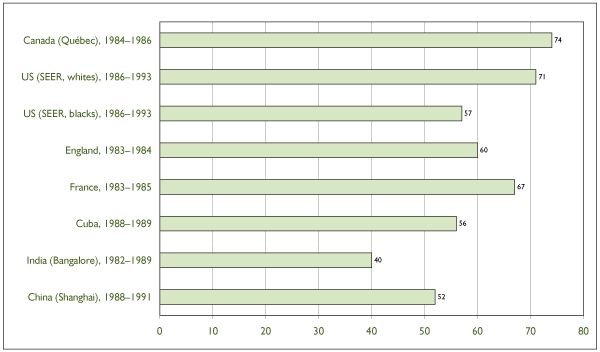
Five-Year Relative Survival Rates Following a Diagnosis of Invasive Cervical Cancer in Canada (Quebec Used as Example), in the United States (SEER Registries) and in Selected European and Developing Countries. Source: Ministère de la santé et des services sociaux du Québec; SEER program; National Cancer Institute of Canada; International Agency for Research on Cancer.

Figure 6 shows the five-year relative survival rates by time since cervical cancer diagnosis for Quebec – as representative of the Canadian experience – for white and Black women in the United States, and for selected developed and developing countries with comparable cohort treatment and follow-up periods. The survival rate was highest among Quebec patients. In North America, patients whose condition is diagnosed and treated in Quebec have had better long-term survival than those in the United States, regardless of ethnicity. The five-year survival rate for Quebec is also among the highest internationally.

Three main factors influence the magnitude of the survival rates: (i) the relative proportions of patients with advanced versus early disease stage, (ii) the age distribution of the cohort of patients, and (iii) access to the main treatment modalities for cervical cancer (i.e. surgery, radiation therapy and chemotherapy) and their combination integrated into specific treatment protocols.[7] These three factors, particularly (i) and (iii), are strongly correlated with socio-economic status. Women of lower economic means may have their diagnosis delayed, which may lead to more advanced disease at the time of treatment and consequently to poorer survival. In addition, they may not be accessing the most modern and effective cancer treatment protocols.

Ability to pay for health care could be a determinant of the substantially different survival rates between white and Black women in the United States and, in specific situations, between the United States and Canada. A recent study comparing cancer survival in Detroit and Toronto found that socio-economic status was associated with cervical cancer survival in Detroit but not in Toronto. Furthermore, the survival rate among the poorest patients in Toronto was significantly better than that among the poorest ones in Detroit for most types of cancer, including cervical cancer.[8] Nevertheless, even in Canada there is a noticeable inverse trend in cervical cancer survival as a function of community income.[9,10]

### Risk Factors

#### The Role of HPV

HPVs are small, double-stranded DNA viruses. As infectious agents, they are highly specific to their respective hosts. More than 120 different HPV types, derived from DNA sequence homology, have been catalogued so far.[11]

Clinical, sub-clinical and latent HPV infections are the most common sexually transmitted viral diseases today,[12] with a peak in prevalence among young women soon after the onset of sexual activity.[12,13] Latent genital HPV infection can be detected in 5% to 40% of sexually active women of reproductive age.[14] In most cases, genital HPV infection is transient or intermittent. [15-17]

In epidemiologic studies conducted during the past 10 years, the relative risks (RRs) for the association between HPV infection (detected by viral DNA testing) and risk of cervical cancer are high – in some studies greater than 100.[18,19] No other risk factor for cervical neoplasia is of comparable magnitude. In fact, few associations in cancer research are as strong as that between HPV and cervical neoplasia, notable exceptions being the link between heavy smoking and lung cancer, and chronic hepatitis B infection and liver carcinoma.[20]

Today, it is well established that infection with the HPV types associated with high oncogenic risk (types 16,18,31,33,35,39,45,51,52,56,58,59 and 68) is the central causal factor in cervical cancer.[14,21] It may even be a necessary cause of this disease and its precursors.[22,23] HPV infection should be considered as a risk exposure, however, since most women who engage in sexual activity will probably acquire HPV infection over a lifetime. The vast majority of these infections will be transient, only a small proportion becoming persistent. A substantial increase in the risk of CIN exists for women who develop persistent, long-term infections with oncogenic HPV types as definedabove.[24,25]

#### Behavioural and Lifestyle Characteristics

##### Sexual Behaviour

See "The Role of HPV." Other prominent risk factors are the role of two measures of sexual activity, namely, number of sexual partners and age at first intercourse, and the sexual behaviour of the woman's male partner(s).[13,26,27]

##### Smoking

Tobacco smoking has consistently emerged as a risk factor for cervical cancer.[28] A direct carcinogenic action on the cervix is conceivable, since nicotine metabolites can be found in the cervical mucus of smokers.[29] Another plausible mechanism is suppression of the local immune response to HPV infection.[12,30] However, a clear assessment of the association is confounded by other variables. Since smoking is associated with sexual behaviour it cannot be easily determined whether its association with cervical neoplasia is genuine or spurious. Studies that have controlled for the effects of age at first intercourse and number of sexual partners have generally found an independent role for tobacco smoking in cervical neoplasia, reporting RRs among current versus never smokers in the range of 1.5–4.5, and evidence of a trend with number of cigarettes smoked and duration of smoking.[31] On the other hand, a few studies have failed to find an association with cigarette smoking.[32,33]

##### Parity

The number of live births per woman is a consistent risk factor for cervical cancer. There is a linear trend in the parity-risk association, as seen in large studies in North America and in Latin America.[34,35] It is possible that multiple pregnancies have a cumulative traumatic or immunosuppressive effect on the cervix, thereby facilitating the acquisition of HPV infection.[36] Another non-mutually exclusive mechanism is the pregnancy-induced hormonal effect on the cervix, which could affect HPV genome elements that are responsive to progesterone.[37]

##### Oral Contraceptive Use

An increased risk of cervical cancer among oral contraceptive (OC) users is found mainly among long-term users. The plausibility of the association rests on the potential for hormonal effects on HPV-containing cervical cells, as it has been shown that steroid stimulation may trigger viral oncogene-related events that could culminate in integration of the virus into the host's genome.[37] Confounding factors are that women who use contraception tend to be more sexually active than those who do not, and women using OC are less likely to use barrier methods of contraception, which have been shown in some studies to exert a protective effect against CIN [38-40] and cervical cancer.[25] It is also possible that some associations may be due to detection bias, since OC users undergo more frequent gynecological examinations than non-users, thereby enhancing detection of early disease.[41]

##### Dietary Factors

High intake of foods (fruits and vegetables) containing carotenoids and vitamin C and, to a lesser extent, intake of vitamins A and E seem to reduce the cervical cancer risk.[42] The results of dietary surveys have been corroborated by assays of plasma micronutrient levels. There is biological plausibility for a protective effect of diet in cervical neoplasia. Carotenoids, tocopherols and ascorbic acid are potent antioxidants that can quench intracellular reactive radicals, thus potentially preventing DNA damage. Beta-carotene, in particular, serves as a metabolic precursor to retinoic acid, which acts by modulating epithelial cell growth and differentiation. Dietary factors may also have a role in cervical immunity.[12] Randomized controlled trials of dietary supplementation to prevent CIN have been conducted or initiated in different populations.

##### Human Immunodeficiency Virus Infection

Patients infected with HIV are prone to develop a variety of infections attributed to their debilitated immune system. HIV infection impairs cell-mediated immunity, thus increasing the risk of HPV-associated diseases, such as genital warts and malignancies. Latent HPV infection and SIL are much more common among HIV-infected women than HIV-negative women from the same populations.[43,44] HPV and HIV infection seem to interact synergistically to increase the risk of CIN, with some further mediation by the degree of immunosuppression.[45] With the successful adoption of antiretroviral therapy in the last few years women are surviving longer with their HIV disease. Little is known, however, about the potential impact of HIV therapy on the natural history of cervical neoplasia among HIV-infected women.

### Differences Between Histological Types

Squamous and adenocarcinomas present many characteristics in common; however, there is also evidence to show that these types may actually have distinct causes despite being so close together anatomically. Most risk factors are indeed common to both types. Four main features differentiate their epidemiologic and prevention characteristics:

1. unlike squamous carcinomas, the incidence of adenocarcinomas has been increasing in recent years, particularly among younger women in Canada[46] and other developed nations;[47]

2. HPV 16 is the HPV type most frequently found in squamous carcinomas, whereas HPV 18 is found in more than half of adenocarcinomas;[48]

3. increased parity is associated with an increased risk of squamous but not adenocarcinomas;[49]

4. Pap cytology (mentioned below) is not as efficacious in detecting adenocarcinomas.

The implications of such discrepancies are yet to have an impact on policy recommendations because current screening recommendations are based on Pap cytology. New screening technologies need to take into account the need to incorporate more sensitive methods for detecting adenocarcinomas.

### Primary Prevention

#### Behaviour Modification

Primary prevention of cervical cancer can be achieved through prevention and control of genital HPV infection. Health promotion strategies geared to a change in sexual behaviour and targeting all sexually transmitted infections of public health significance can be effective in preventing HPV infection.[50,51] Although there is consensus that symptomatic HPV infection (genital warts) should be managed by treatment, counselling, and partner notification, active case-finding of asymptomatic HPV infection is currently not recommended as a control measure.

#### Immunization Against HPV

Two main types of HPV vaccine are currently being developed: prophylactic vaccines to prevent HPV infection and associated diseases, and therapeutic vaccines to induce regression of precancerous lesions or remission of advanced cervical cancer. Such vaccines are already under evaluation in phase I and II trials in different populations.[52] Immunization against HPV may have greatest value in developing countries, where 80% of the global burden of cervical cancer occurs each year and where Pap screening programs are less likely to be effective. At present, it is difficult to speculate about the direction of research in this area. Although the preliminary results from phase II trials of prophylactic vaccines have been successful, it will take many years before vaccines can be assessed as a cervical cancer prevention strategy.[53]

### Secondary Prevention

Cervical cancer screening is currently one of the most active areas of research in cancer prevention (see "Other Cytology Methods" below). Several new technologies are under evaluation, and professional and government groups are considering their contribution in a reassessment of practice guidelines currently being undertaken.

#### Pap Cytology Screening

Efficacy of Pap Cytology Screening: There have been no controlled trials of Pap screening efficacy, either randomized or not. The evidence for the efficacy of Pap smear screening in cervical cancer comes mainly from three sources: (i) epidemiologic studies reporting a risk of invasive cervical cancer 2–10 times greater among women who have not been screened and an increased risk with time since last normal smear or with lower frequency of screening; (ii) cervical cancer incidence and mortality rates, which decreased sharply following the introduction of cytology screening in Scandinavian countries, Canada and the United States, and did so in proportion to the intensity of the screening efforts; and (iii) multiple national and international consensus panels worldwide.[31]

In spite of its success, cytology has important limitations, false negative results being the most significant. One recent meta-analysis indicated that the average sensitivity of a single Pap test to detect HSIL or cervical cancer was 51%, whereas its specificity was 98%.[54] The solution to minimizing false-negative errors in cytology is to improve the quality of smear taking, slide processing and overall diagnostic performance of cervical cytology. False-negative diagnoses have important medical, financial and legal implications.

##### Canadian Practices

The Canadian Task Force on Preventive Health Care[55] and a series of consensus workshops [56-59] have provided uniform national recommendations that have been reaffirmed on separate occasions by independent provinces or by cancer prevention coalitions across the country. The Cervical Cancer Prevention Network, created as an informal association of federal and provincial representatives and clinical professional bodies, is the most important of these coalitions.

In essence, the prevailing guidelines recommend that Pap screening is to begin at age 18 or at initiation of sexual activity and to continue annually. After two negative consecutive smears, one year apart, screening is to proceed every three years to age 69. The national workshop recommendations also state that this screening schedule be implemented in combination with an efficient information system allowing rapid case notification and recall.[56] The empirical basis for the soundness of this set of management guidelines was recently published.[60]

#### Other Cytology Methods

There are several automated systems being tested and marketed. In one of these, liquid-based cytology, the sample recovered from the cervix is suspended in a cell-preserving solution rather than placed on a glass slide. Excess blood and inflammatory cells are lysed, and approximately 50,000 diagnostic cells are randomly transferred by the equipment as a thin layer onto a glass slide by a robotic cell processor. The slides are stained and then read by cytotechnologists. Results from clinical studies have shown that automated thin-layer slides can improve detection of atypical cells, precursor lesions and cancer by producing uniformly cleaner slides free of blood, debris and cell clumps that interfere with microscopic reading.[27,61] A recent meta-analysis concluded that liquid-based cytology had superior sensitivity and equivalent specificity to conventional cytology, and economic models have indicated that it could lead to lower cost per life-year saved in the United States.[62]

Computer-assisted scanners map the smear in order to detect abnormal cells, thereby separating any slides that contain suspect images for subsequent reading by a cytotechnologist. A key advantage is the potential to alleviate the shortage of qualified workers in cytopathology. Comparative trials, mostly funded by the private sector, are taking place in many laboratories in North America and Europe to answer questions related to screening efficacy and the cost-effectiveness of automated devices.

#### Screening by HPV Testing

Since the mid-1990s there has been substantial interest in the use of standardized HPV DNA testing as a cervical cancer screening tool under the premise that it will provide acceptable diagnostic performance while being more reproducible and more easily adapted for clinical practice than conventional Pap cytology. Several studies have assessed the test's diagnostic performance (for high-risk types) in a variety of populations, using cross-sectional or short-term follow-up rather than more extended review of incidence or mortality rates. [63-69] Lesion definition varied across studies and included either CIN of all grades or CIN 2 or 3 or worse lesions.

HPV DNA testing has been shown to have, on average, a 25% greater sensitivity than Pap cytology but somewhat lower specificity (on average, 10% lower) for detecting CIN 2/3 or cancer. Screening of women aged 30 or older tended to improve the specificity considerably, because viral infections in this age group are less likely to be of a transient nature than those in younger women. An important finding of most studies was that the combination of cytology and HPV testing attained very high negative predictive values (approaching 100%), which, at least in theory, could safely permit longer screening intervals, thus lowering costs. Definitive evidence of efficacy is still needed from long-term follow-up studies with invasive cancer as an outcome and from randomized controlled trials.

One other screening application of HPV testing is in the secondary triage of equivocal Pap smears. Results from two large-scale studies have indicated that HPV testing has greater sensitivity than a repeat Pap test for detecting hidden HSIL or cancer among women referred because of an equivocal Pap smear. At the same time it results in reduced costs in terms of colposcopy referrals.[70,71]

## Discussion

### Data Limitations

There is a lack of Canadian data on cervical cancer in women from different ethnic backgrounds, and this precludes preventive efforts targeted at vulnerable groups.

Most cases of cervical cancer occur because of a false-negative result of the Pap test, or because the woman did not receive a Pap test at all or did not receive it often enough. Well-developed recommendations and guidelines exist as part of a succession of national consensus reports concerning the implementation and maintenance of cytology-based screening programs. However, most Canadian women do not yet have access to organized, centralized cervical cancer screening.

Despite the availability of some excellent Canadian surveys of Pap test utilization among women who developed the invasive form of the disease, we still lack critical information concerning the appropriate management of precursor lesions from audit studies. Various automated systems aimed at improving the performance of the cytology test are currently being tested. Large-scale prospective studies are still needed to evaluate these systems.

### Recommendations

There have been several studies assessing the relative utility of HPV testing in addition to or compared with the Pap test as a cervical cancer screening tool; these have been cross-sectional or short-term follow-up investigations, and no randomized controlled trials have yet been published. HPV testing seems to be a promising screening approach, but definitive evidence of efficacy is still needed from long-term follow-up studies with invasive cancer as an outcome and from randomized controlled trials. One other screening application for HPV testing is in the secondary triage of equivocal Pap smears. Results from large-scale studies have indicated that HPV testing has greater sensitivity than a repeat Pap test for detecting hidden precursor lesions or cancer among women referred because of an equivocal Pap smear, while resulting also in reduced costs for colposcopy referrals. However, taking into account the totality of the evidence, we still lack Canadian data to justify augmenting or modifying current screening programs.

## Note

The views expressed in this report do not necessarily represent the views of the Canadian Population Health Initiative, the Canadian Institute for Health Information or Health Canada.
